# Does spending matters? Re-looking into various covariates associated with Out of Pocket Expenditure (OOPE) and catastrophic spending on accidental injury from NSSO 71st round data

**DOI:** 10.1186/s13561-017-0177-z

**Published:** 2017-12-20

**Authors:** Jalandhar Pradhan, Rinshu Dwivedi, Sanghamitra Pati, Sarit Kumar Rout

**Affiliations:** 1 Department of Humanities and Social Sciences, National Institute of Technology, Rourkela, Orissa 769 008 India; 2 Regional Medical Research Centre, Bhubaneswar, Odisha 751023 India; 3 Indian Institute of Public Health-PHFI, Bhubaneswar, Odisha 751024 India

**Keywords:** Accidental, Socioeconomic, OOPE, Logistic, Economic burden

## Abstract

**Background:**

Accidental Injury is a traumatic event which not only influences physical, psychological, and social wellbeing of the households but also exerts extensive financial burden on them. Despite the devastating economic burden of injuries, in India, there is limited data available on injury epidemiology. This paper aims to, first, examine the socio-economic differentials in Out of Pocket Expenditure (OOPE) on accidental injury; second, to look into the level of Catastrophic Health Expenditure (CHE) at different threshold levels; and last, to explore the adjusted effect of various socio-economic covariates on the level of CHE.

**Methods:**

Data was extracted from the key indicators of social consumption in India: Health, National Sample Survey Organisation (NSSO), conducted by the Government of India during January–June-2014. Logistic regression analysis was employed to analyse the various covariates of OOPE and CHE associated to accidental injury.

**Findings:**

Binary Logistic analysis has demonstrated a significant association between socioeconomic status of the households and the level of OOPE and CHE on accidental injury care. People who used private health services incurred 16 times higher odds of CHE than those who availed public facilities. The result shows that if the person is covered via any type of insurance, the odd of CHE was lower by about 28% than the uninsured. Longer duration of stay and death due to accidental injury was positively associated with higher level of OOPE. Economic status, nature of healthcare facility availed and regional affiliation significantly influence the level of OOPE and CHE.

**Conclusion:**

Despite numerous efforts by the Central and State governments to reduce the financial burden of healthcare, large number of households are still paying a significant amount from their own pockets. There are huge differentials in cost for the treatment among public and private healthcare providers for accidental injury. It is expected that the findings would provide insights into the prevailing magnitude of accidental injuries in India, the profile of the population affected, and the level of OOPE among households.

## Introduction

Injuries^1^ are well acknowledged globally as a major cause of death and disability, and road traffic injuries (RTI)^2^ accounted for nearly more than 1 million deaths in 2015 [1–3]. As per WHO estimates, RTI, are predicted to be the fifth leading cause of death in all age groups by the year 2030. Injuries results into more annual deaths as compared to HIV, Malaria, and Tuberculosis combined [4]. Each year approximately 250 million people worldwide are affected by injury, and of these more than 300,000 people die [5]. The socioeconomic burden of injury is quiet significant which affects most adversely to the younger and most productive demographic segment of the population [6]. In particular, the burden is disproportionately in Low and Middle-Income Countries (LMICs) where more than 90% of the mortality occurs due to unintentional injuries, primarily due to Road Traffic Accidents (RTA)^3^ [7, 8]. Individuals who sustain injuries in LMICs are six times more likely to die than those in High-Income Countries (HICs), given the limited capabilities for trauma care in low-income settings [9].

Within LMICs, India faces one of the highest burdens of injuries. According to a study on the burden of diseases and injuries, 28% of years of life lost (YLL) in India are attributed to injuries [10]. In fact, injuries are the first cause of YLL among all causes of death in India. As per the World Health Organization (WHO), injury is the second leading cause of death in India, with deaths annually from RTA being among the highest in the world [4, 11]. During the past few years, with the growing pattern of industrialization and motorization, there has been an increase in the number of deaths from injuries [12–14]. This is projected to escalate further since the country is undergoing rapid urbanization. Even though the burden of diseases in India has been aggravated by accidental injuries, limited information is available about the economic consequences associated with it. The direct cost involved in the accidental injury was found to be huge, which not only forces the households into the poverty trap due to high treatment cost and disability, but also imposes high economic as well as societal cost too [15, 16]. Estimating the cost of injuries is identified among the five priority items to address the global burden of unintentional injuries [17].

## Background

Indian health system is characterized by low public spending, as major share of the health expenditure is borne by the households in form of out-of-pocket expenditures (OOPE)^4^ [18–20]. In case of accidental injury, households have to bear heavy cost both in terms of direct as well as indirect health expenditure. The impoverishment of households in India due to OOPE and the catastrophic health expenditure (CHE)^5^ on health are well documented [16, 21, 22] and these are reported to be higher for injuries than for other ailments [17]. The majority of India’s citizens receive health care through a publicly funded system governed by the Ministry of Health. Data from public health facilities may provide some insights on injuries, however, many of the injuries could have been reported to non-public or private health care facilities or not been reported at all [23]. Further, hospital records, albeit helpful to understand patterns and mechanisms of injury retrospectively, have been found to be deficient in several settings [24]. Few previous works on a tertiary hospital had identified that fundamental information on patient demographics, circumstances under which accidents^6^ occur; treatment costs and modalities were frequently not recorded [25].

Despite the overwhelming burden of injuries, there is limited data on injury epidemiology and its outcomes in India. Mortality statistics, though used as an indicators of injury magnitude, may represent the ‘tip of the iceberg’ since non-fatal injuries exceed fatal injuries by up to 20 times [26, 27]. As such no nationwide data is available on the prevalence, demographic profile of the injured persons, treatment and outcome of the accidental injury in the country. Available studies are confined either to the single facility or they are limited to some specific category such as RTA, which fails in providing complete picture of the injury profile in India [24, 27]. Public health interventions and policies to reduce harm and prevent accidents would be effective only when they are designed for the right population, at the right time and in the right setting [28].

Since cost of injuries may vary according to age and gender, influenced by subtle variation of cultural lifestyle and behavioral pattern, knowledge of trauma epidemiology is essential for identifying the correlates of OOPE on accidental injuries and development of solutions from the public health viewpoint. Accidental injury involves higher expenses on seeking healthcare services and forces households to spend significantly higher amount from their own pockets leading to impoverishment. As per available information in the latest NSSO report (NSS KI(71/25.0, NSSO, 2014), [17] average medical expenditure per hospitalization case on accidental injury, is the fifth highest expenses borne by the households after OOPE on other major ailments such as cancer, cardio-vascular, genitourinary, and psychiatric and neurological diseases (Appendix 1). Though injury is an important cause of death in India, there is limited knowledge regarding the economic impact on households. Earlier, studies examining the economic impact of injury on households were based on either hospital settings or on the basis of limited samples. This is not adequate to explain the overall economic impact of injury on households. Given the limitations of previously reported studies, we undertook a study to determine the magnitude of injuries, their distribution and associated health care expenditure in India. The major objectives of this paper are: first, to describe the prevalence of accidental injury among the sampled population, second, to examine the socio-economic differentials in OOPE on accidental injury by taking both direct and indirect costs into account; third, to look into the level of CHE (5%, 10% and 15%) at various threshold levels due to accidental injury; fourth, to measure the adjusted effect of various covariates on the level of CHE. It is expected that the findings would provide insights into the prevailing magnitude of accidental injuries in India, indicate the epidemiological distribution, the profile of the population affected and correlates and identify the prevalence of catastrophic expenditure, if any. Such breadth of information could be used to inform the planning central to all facets of injury prevention.

## Method

Data is used from the key indicators of social consumption in India: Health**,** National Sample Survey Organization (NSSO), conducted by the Government of India January–June-2014 i.e., the 71st round. The present study uses household schedule 25.0 questionnaires. The Socio-economic Survey (SES) rounds contain information on household’ssocial consumption of health on various heads such as, the proportion of ailing persons, spells of ailments and their treatment, rate of hospitalization, the cost of treatment-hospitalization and the cost of treatment–non-hospitalized. The recall periods of institutional expenses are for 365 days [17]. Here we have included cost under two heads i.e., direct and indirect. Direct cost includes doctor’s/surgeon’s fee (hospital staff/other specialists, medicines, diagnostic tests, bed charges, other medical expenses (attendant charges, physiotherapy, personal medical appliances, blood, and oxygen). Indirect cost includes the cost of transport for the patient, other non-medical expenses (in INR) incurred by the households (food, transport for others, expenditure on escort, lodging charges if any, etc.). The outcome variable for the study is OOPE on injury. The approach to measure OOPE for healthcare payments was adopted from Wagstaff and Doorslaer, in the World Bank document. In addition to the medical and non-medical expenditure, information was also available on the household’s socio-economic and demographic characteristics.

### Variables under study

This paper explains the association between the HE and socio demographic variables and how this changes across caste, education and other SES variables. The independent variables (relating to of the individuals and households) in the study are: age (less than 15, 15–29, 30–59 and 60+); sex (male and female); level of education (illiterate, up to primary, up to secondary, graduation and above); place of residence (urban/rural); social group (Scheduled Caste (SC), Scheduled Tribe (ST), Other Backward Caste (OBC) and Others); religious affiliation (Hindu, Muslims and Others); household size (1–3 members, 4–6 and 7+ members); economic status (Poorest, Poorer, Middle, Richer and Richest); level of care (public/private); insurance coverage (covered/not covered); source of financing (income/savings, borrowings and others); survival status (Dead/Alive); duration of stay (1–2 days, 3–7 days, 8–14 days and 14+ days); and, region (north, northeast, east, central, west, south).

First, descriptive analysis was done to assess the socio-economic and demographic profile of the persons with accidental injury. Second, we calculated socio-economic differentials in OOPE (with 95% CI) on the accidental injury. Third, CHE was calculated at different threshold levels (5%, 10%, and 15%). Last, Binary logistic regression analysis was employed to explore the relative effect of socio-economic and demographic characteristics on the level of CHE for injury in India.

Logistic regression can be used to predict a dependent variable on the basis of independents, and to determine the percent of the variance in the dependent variable explained by the independents; to rank the relative importance of independents; to assess interaction effects, and to understand the impact of covariates. Logistic regression applies maximum likelihood estimation after transforming the dependent into a logit variable (the natural log of the odds of the dependent occurring or not). So, logistic regression estimates the probability of certain event, whether occurring or not. The multiple logistic models can be noted as:

$$ \ln \left(\frac{p}{1-p}\right)=\alpha +{\beta}_1{x}_1+{\beta}_2{x}_2+{\beta}_3{x}_3+\dots {\beta}_i{x}_i+e $$

Where, *p* is the probability of occurrence of multimorbidity, *p(у = 1);β*_1_ *β*_2_, *β*_3_,… *β*_*i*_ refer to the beta coefficients;*x*_1_ *x*_2_ *x*_3_ ….*x*_*i*_ Refer to the independent variables and e is the error term. STATA 12 is used to analyze the data. We have also looked into the problem of endogeneity, which may not arise in this case due to following reasons. As we all know the problem of endogeneity occurs due to measurement errors and omitted variable bias. The measurement errors could be due to recall bias and other similar issues related to the explanatory variables. This problem is addressed in a large sample here. Further, the measurement errors will not arise here as whatever is true for higher caste for instance, will hold good for the lower caste or whatever is true for lower education will also hold good for higher education. It is because the explanatory variables are not systematically different.

## Results

### Socio-demographic profile of the respondents with accidental injury

Socio-economic and demographic characteristics of the persons with accidental injury^7^ (Table 1) indicates that about 48% belonged to the age group of 30–59 years, 24% were in age group of 15–29, 16% were aged 60+ and 12% were in the age group of less than 15 years. About 56% belong to rural background. Nearly 73% of the persons with accidental injury were males. About, 24% sampled population were illiterate, 26% had studied up to primary, 39% up to secondary, and 12% had studied up to graduation and above. Majority of the persons belonged to OBC group, followed by Others, SCs and STs. Nearly 80% persons belonged to the Hindu community, 12% were Muslims and 8% were of other religious groups.

**Table 1:** Socio-economic and demographic characteristics of persons with accidental injury, India, 2014

Co-variates	Percent	Number
Age		
less than 15	11.9	482
15–29	24.3	985
30–59	47.7	1934
60+	16.1	654
Sex		
Male	73.3	2973
Female	26.7	1082
Education level^a^		
Illiterate	24.1	941
Up to primary	25.9	1011
Up to secondary	38.6	1509
Graduation and above	11.5	449
Residence		
Rural	55.8	2264
Urban	44.2	1791
Caste		
Scheduled Tribes	9.5	387
Scheduled Castes	17.9	724
Other Backward Classes (OBC)	39.3	1595
Others	33.3	1349
Religion		
Hindu	79.6	3227
Muslim	12.2	493
Others	8.3	335
Household size		
1–3 member	21.0	853
4–6 members	57.6	2335
7 + members	21.4	867
Wealth quintile		
Poorest	15.0	608
Poorer	20.9	845
Middle	19.4	788
Richer	23.0	935
Richest	21.7	879
Level of care		
Public	41.2	1670
Private	58.8	2385
Insurance coverage^a^		
Not covered	79.2	3098
Covered	20.8	812
Reimbursement status		
Received	4.3	174
Not received	95.7	3881
Survival status		
Alive	96.4	3910
Dead	3.6	145
Duration of stay		
1–2 days	24.3	985
3–7 days	39.4	1599
8–14 days	17.0	688
14+ days	19.2	783
Source of financing		
Household income and saving	69.6	2826
Borrowings	23.0	933
Other sources	7.3	296
Regions^b^		
North	15.5	628
Northeast	7.7	312
East	17.2	697
Central	22.4	909
West	13.1	531
South	24.1	978
Total	100	4055

Source: Authors’ estimates from NSSO survey, 2014

^a^Based on 3910 cases

^**b**^ **North**: J& K Himachal Pradesh, Punjab, Chandigarh, Haryana, Delhi, Rajasthan; **Northeast:** Sikkim, Arunachal Pradesh,, Nagaland, Manipur, Mizoram, Tripura, Meghalaya, Assam; **East:** Bihar, West Bengal, Jharkhand, Odisha; **Central:** Uttar Pradesh, Chhattisgarh, Madhya Pradesh; **West:** Gujrat, Daman and Diu, Dadar and Nagar Haveli, Maharashtra, Goa; **South:** Andhra Pradesh, Karnataka, Lakshadweep, Kerala, Tamil Nadu, Pondicherry, Andaman and Nicobar, Telangana

About 59% people were availing private facilities; others depended on public care. Insurance coverage for accidental injury was low since only 21% were covered by any type of insurance scheme; about 79% were not covered by any insurance scheme. Nearly 96% of the households had not received any reimbursement from the insurance providers. Survival status indicates that 96% people who suffered from accidental injury survived while 4% died. Nearly 39% sampled population stayed in the hospital for an average of 3–7 days, followed by other categories. A major source of financing for accidental injury was from household income and savings; nearly 70% financing was from own pocket while 23% resorted to borrowings and 7% managed financing from other sources. Regional distribution of the sampled population, who had faced accidental injuries, was higher in the Southern (24%) region followed by Central (23%), East (17%), Northern region (16%) and others. However the share of accidental injuries out of the total sampled general population by selected socio-economic and demographic covariates was not that higher (1.21% out of general population) (Appendix 2).

### Socio-economic differentials in the level of OOPE

Table 2 shows the socioeconomic differentials in OOPE. We have first analyzed the pattern of OOPE (medical, transportation and non-medical), without taking into consideration the total amount of reimbursement from insurance. Later, for examining the effect of reimbursement on the total OOPE, the amount reimbursed was deducted from the total expenditure on accidental injury care. The share of mean OOPE (without considering reimbursement amount) was higher for medical expenses (INR 24916) followed by non-medical expenditure (INR 1909) and transportation (INR 906), in the overall accidental injury expenditure (INR 27731). People in the age group of 60+ had spent more under all heads i.e., medical care (INR 30567), non-medical (INR 2376) and transportation (INR 1097), in comparison to other age groups. Similarly, spending on accidental injury among males was higher than for females under all the heads.

**Table 2:** Socio-economic differentials in OOPE (in INR), India, 2014

Co-variates	Medical	Transport	Non-medical	Total (Including reimbursement amount)	Total (Excluding reimbursement amount)
Age					
less than 15	12,822	589	1234	14,646	14,165
15–29	24,535	937	1908	27,380	25,782
30–59	26,606	914	1938	29,457	27,224
60+	30,567	1097	2376	34,040	33,309
Sex					
Male	26,173	988	2094	29,255	27,542
Female	21,337	674	1380	23,391	22,116
Education level^a^					
Illiterate	18,782	785	1568	21,135	20,849
Up to primary	20,182	906	1832	22,920	22,248
Up to secondary	28,415	878	2084	31,377	29,367
Graduation and above	38,660	930	2000	41,589	34,851
Residence					
Rural	21,930	979	1863	24,772	24,127
Urban	30,691	765	1997	33,453	30,008
Caste					
Scheduled Tribes	12,589	762	1774	15,126	14,917
Scheduled Castes	19,978	791	1822	22,591	21,537
Other Backward Classes (OBC)	24,130	891	1872	26,894	26,073
Others	31,507	1030	2042	34,579	31,243
Religion					
Hindu	26,126	927	2014	29,066	27,597
Muslim	18,250	779	1234	20,263	17,858
Others	23,429	909	1987	26,325	24,752
Household size					
1–3 member	25,892	846	1903	28,640	25,909
4–6 members	23,716	890	1827	26,433	24,887
7 + members	27,871	1025	2185	31,081	30,486
Wealth quintile					
Poorest	15,579	1116	1524	18,219	18,086
Poorer	18,355	770	1462	20,587	20,446
Middle	18,460	746	1570	20,775	19,110
Richer	25,312	799	2023	28,134	27,113
Richest	47,496	1217	3008	51,720	46,467
Level of care					
Public	7014	746	1426	9186	8869
Private	36,261	1008	2215	39,483	37,071
Insurance coverage^a^					
Not covered	24,466.00	881.00	1861	27,208	27,049
Covered	25,565.00	826.00	1936	28,327	21,722
Reimbursement status					
Received	66,720	1223	22,167	70,159	24,691
Not received	23,392	895	1897	26,184	26,184
Survival status^a^					
Dead	29,365	1794	2605	33,764	33,661
Alive	24,723	868	1878	27,469	25,805
Duration of stay					
1–2 days	4858	354	400	5613	5422
3–7 days	18,638	842	1320	20,801	19,143
8–14 days	32,199	1181	2433	35,813	34,170
14+ days	60,380	1595	4833	66,809	63,385
Source of financing					
Household income and saving	20,163	727	1528	22,419	21,157
Borrowings	35,378	1260	2780	39,400	36,973
Other sources	29,573	1231	2225	33,031	31,418
Regions^b^					
North	23,729	1046	1990	26,765	24,196
Northeast	8970	683	1329	10,982	10,641
East	19,256	1038	1555	21,850	19,776
Central	25,560	816	1671	28,048	27,654
West	35,611	905	1928	38,443	36,187
South	24,520	836	2231	27,586	26,203
Total	24,916.	906	1909	27,731	26,132

Source: Authors’ estimates from NSSO survey, 2014

^**a**^Based on 3910 cases

^**b**^ **North**: J& K Himachal Pradesh, Punjab, Chandigarh, Haryana, Delhi, Rajasthan; **Northeast:** Sikkim, Arunachal Pradesh,, Nagaland, Manipur, Mizoram, Tripura, Meghalaya, Assam; **East:** Bihar, West Bengal, Jharkhand, Odisha; **Central:** Uttar Pradesh, Chhattisgarh, Madhya Pradesh; **West:** Gujrat, Daman and Diu, Dadar and Nagar Haveli, Maharashtra, Goa; **South:** Andhra Pradesh, Karnataka, Lakshadweep, Kerala, Tamil Nadu, Pondicherry, Andaman and Nicobar, Telangana

Those who were educated till graduation or above were spending the highest on medical, transportation and non-medical heads compared to others. People residing in the urban areas were spending higher than their rural counterparts on medical, non-medical, and in terms of overall expenses. Other caste people were spending higher than their OBC, SC and ST counterparts under all the heads. Results indicate that Hindus were spending more than *others* and *Muslims* in accidental injury cases. People belonging to the richest wealth quintile were spending maximum under all the heads, followed by others. Minimum expenditure was among people who belong to the poorest economic status. Similarly, expenses were higher if the accidental injury care was sought from private facility and with access to some sort of insurance coverage. Average expenditure under all the heads was higher for deceased persons in comparison to those who had survived accidental injury. With the lengthening of stay duration in the hospital, OOPE increased at a significant rate. The level of OOPE was higher among households who met their healthcare financing need through borrowings [29]. Results also indicate regional variations in the mean OOPE - spending was higher in the Western region, followed by Central and Southern regions. Lowest level of OOPE was recorded among the Northeastern states.

Finally, we presented the socio-economic differentials in OOPE excluding the amount of reimbursement. Results suggest that the mean OOPE was marginally lower for households who received reimbursement (INR 26,132), compared to their counterparts (INR 27731). The reimbursed amount was higher among elderly population, males, households educated above graduation, residing in the urban areas, and belonging to the others as caste group. Similarly, Muslims, households with 1–3 members, higher economic section, seeking private care covered via insurance schemes and received reimbursement, stayed in the hospital for more than 14 days, received higher reimbursement, which marginally reduced the burden of OOPE as compared to other categories. However, even after receiving reimbursement from insurance companies, the households were paying a significant amount from their own pockets for seeking care for accidental injury.

### Level of catastrophic health expenditure on accidental injury care

Table 3 shows the CHE at 5%, 10% and 15% level for accidental injury care. Results indicate that at 5% level, CHE was highest among categories -persons in the age group of 15–29 years, males, attained education up to graduation or above, residing in the rural areas, belongs to SC and Other social group, other religious communities, household size up to 1–3 members and belongs to the poorest section of the population.

**Table 3:** CHE at 5, 10 and 15% level, India, 2014

Co-variates	5%	10%	15%
Age			
less than 15	70.1	48.1	34.6
15–29	75.0	56.9	45.1
30–59	71.9	55.2	44.6
60+	72.5	58.2	49.0
Sex			
Male	74.4	56.7	45.7
Female	67.5	51.4	40.6
Education level^a^			
Illiterate	71.3	56.7	45.9
Up to primary	71.6	53.4	41.5
Up to secondary	73.1	54.8	44.5
Graduation and above	75.8	57.8	44.8
Residence			
Rural	75.2	57.8	46.3
Urban	69.2	52.1	41.7
Caste			
Scheduled Tribes	69.2	47.2	34.1
Scheduled Castes	73.1	57.1	46.2
Backward Classes	72.7	54.9	44.8
Others	73.1	57.0	45.4
Religion			
Hindu	72.8	56.1	45.6
Muslim	69.5	51.5	41.7
Others	73.7	52.8	34.6
Household size			
1–3 member	77.3	62.3	52.2
4–6 members	71.7	53.9	42.5
7 + members	69.9	52.0	41.1
Wealth quintile			
Poorest	79.1	66.1	54.6
Poorer	74.5	57.2	47.6
Middle	74.7	56.1	43.5
Richer	69.1	52.2	41.9
Richest	67.9	48.4	37.0
Level of care			
Public	52.2	32.8	23.4
Private	86.7	71.0	58.8
Insurance coverage^a^			
Not covered	73.9	56.3	45.0
Covered	68.1	51.3	40.8
Survival status			
Dead	68.2	55.8	48.2
Alive	72.7	55.2	44.1
Duration of stay			
1–2 days	44.0	22.0	13.4
3–7 days	74.5	53.6	39.5
8–14 days	86.7	73.9	64.0
14+ days	91.8	84.0	75.4
Source of finance			
Household income and saving	66.1	47.4	36.2
Borrowings	89.7	75.3	64.5
Other sources	79.7	66.8	57.7
Regions			
North	69.1	51.4	39.9
Northeast	70.1	43.9	29.1
East	72.8	55.6	44.6
Central	74.0	57.7	47.5
West	79.8	64.7	53.3
South	69.9	53.7	43.7
Total	72.5	55.3	44.3

Source: Authors’ estimates from NSSO survey, 2014

^a^Based on 3910 cases

Similarly, those who had availed care from private providers, not covered by insurance, survived after the accidental injury, financed from the borrowings, stayed in the hospital more than 14 days, belongs to the western regions, also incurred higher CHE at 5%. At the 10% level, also similar pattern was observed regarding CHE except for a few exceptions such as, older population of 60+ years, households who belong to the Hindu religious community and persons who died after the accidental injury, who incurred higher CHE. At the 15% level, similar trends were recorded in terms of CHE as observed at 10%, on accidental injury except for one exception; here CHE was more concentrated among persons who were illiterate. Rest of the variables showed similar results.

### Results from the logistic regression analysis

Table 4 shows the results of the Binary logistic regression analysis on the related covariates of the determinants of accidental injury at 5%, 10% and 15% levels of CHE. Results reveal that odds of CHE at 5% are about 33% lower for females as compared to males. Educational level shows that persons who are educated up to primary have significantly lower odds of CHE. Odds of CHE for people who were educated up to graduation or above were about two times higher than those who were illiterate. The occurrence of CHE was lower by 44% for Muslims as compared to Hindus. Odds of CHE were significantly lower by 79% and 68% for the richest and richer section respectively, compared to the poorest. People who used private health services incurred 16 times higher odds of CHE than those who availed public facilities. The result shows that if the person is covered via any type of insurance, the odd of CHE was lower by about 28% than the uninsured. Duration of stay is an important determinant of CHE. Results indicate that odds of CHE for accidental injury care was about 34 times higher for those who stayed in the hospital for more than 14 days, as compared to those who stayed for 1–2 days. The analysis shows that odds of CHE at 10% and 15% are about two times higher for people who belong to the age group of 60+ compared to less than 15 years age group. Similarly, odds of catastrophic spending were nearly two times higher for people who had education up to graduation or above. Odds of CHE at 10% and 15% level was 89% and 87% lower for the richest section of the population as compared to the poorest group. Similarly, the odds of CHE was 14 (10% level) and 12 times (15% level) higher in private care in comparison to those who have availed care from government facilities. Odds of CHE at 10% and 15% were 55% and 70% lower for those who survived the accident than the deceased. Duration of stay significantly influences the level of CHE. Odds of CHE at 10% were about 56 times and at 15% it was 60 times higher, for those who stayed in the hospital for more than 14 days, in comparison to those who stayed only for 1–2 days. The results indicate that odds of CHE were three times higher (at 10% CHE) and two times higher (at 15% CHE) for those who borrowed for accidental injury care, than other counterparts. Odds of CHE were nearly 61% lower for the southern region people at 10% CHE and 62% lower at 15% CHE than others.

**Table 4:** Results from logistic regression analysis, India, 2014

Co-variates	5%			10%			15%		
	OR	95% CI		OR	95% CI		OR	95%CI	
Age									
less than 15									
15–29	1.25	[0.73	2.13]	2.02^*^	[1.01	4.04]	1.74^*^	[0.94	3.22]
30–59	0.92	[0.58	1.47]	1.73^*^	[0.94	3.22]	1.72^*^	[1.00	2.96]
60+	1.01	[0.59	1.73]	2.16^*^	[1.06	4.43]	2.20^*^	[1.12	4.31]
Sex									
Male									
Female	0.67^*^	[0.47	0.96]	0.90	[0.66	1.22]	0.98	[0.72	1.32]
Education level^*^									
Illiterate									
Up to primary	0.70^*^	[0.47	1.05]	0.88	[0.59	1.30]	1.04	[0.70	1.56]
Up to secondary	1.21	[0.77	1.89]	1.18	[0.74	1.89]	1.59^*^	[1.00	2.55]
Graduation and above	1.90^*^	[1.04	3.45]	2.19^**^	[1.23	3.89]	2.10^*^	[1.19	3.71]
Residence									
Rural									
Urban	0.80	[0.58	1.11]	0.86	[0.63	1.16]	0.93	[0.69	1.26]
Caste									
Scheduled Tribes									
Scheduled Castes	0.71	[0.38	1.33]	1.05	[0.45	2.45]	0.94	[0.50	1.77]
Backward Classes	0.94	[0.52	1.70]	1.43	[0.61	3.32]	1.25	[0.67	2.34]
Others	1.03	[0.56	1.89]	1.82	[0.79	4.21]	1.54	[0.81	2.94]
Religion									
Hindu									
Muslim	0.56^*^	[0.35	0.89]	0.74	[0.49	1.11]	0.80	[0.53	1.21]
Others	1.01	[0.51	2.00]	0.98	[0.60	1.60]	0.73	[0.43	1.23]
Household size									
1–3 member									
4–6 members	0.95	[0.65	1.38]	0.84	[0.60	1.18]	0.88	[0.62	1.24]
7 + members	0.74	[0.46	1.20]	0.73	[0.46	1.15]	0.75	[0.49	1.17]
Wealth quintile									
Poorest									
Poorer	0.72	[0.44	1.19]	0.45^**^	[0.28	0.72]	0.59^*^	[0.38	0.91]
Middle	0.61^*^	[0.36	1.03]	0.26^***^	[0.15	0.45]	0.24^***^	[0.14	0.41]
Richer	0.32^***^	[0.18	0.54]	0.24^***^	[0.14	0.43]	0.29^***^	[0.17	0.48]
Richest	0.21^***^	[0.12	0.37]	0.11^***^	[0.06	0.20]	0.13^***^	[0.07	0.22]
Level of care									
Public									
Private	16.38^***^	[11.62	23.08]	14.41^***^	[10.34	20.09]	11.91^***^	[8.57	16.57]
Insurance coverage^*^									
Not covered									
Covered	0.72^*^	[0.51	1.03]	0.85	[0.62	1.17]	0.78	[0.55	1.11]
Survival status									
Dead									
Alive	0.65	[0.30	1.41]	0.45^*^	[0.21	0.97]	0.30^**^	[0.15	0.61]
Duration of stay									
1–2 days									
3–7 days	6.90^***^	[4.80	9.91]	6.74^***^	[4.65	9.76]	7.57^***^	[5.23	10.94]
8–14 days	21.23^***^	[13.20	34.16]	25.18^***^	[16.10	39.39]	23.73^***^	[15.42	36.53]
14+ days	33.87^***^	[19.68	58.28]	56.26^***^	[35.46	89.25]	59.85^***^	[37.97	94.34]
Source of finance									
Household income and saving									
Borrowings	4.61^***^	[3.02	7.04]	3.44^***^	[2.48	4.77]	2.33^***^	[1.69	3.22]
Other sources	3.29^**^	[1.56	6.93]	2.22^**^	[1.22	4.05]	2.06^**^	[1.20	3.55]
Regions									
North									
Northeast	1.73^*^	[0.91	3.31]	1.23	[0.64	2.37]	0.84	[0.44	1.59]
East	1.43	[0.87	2.35]	0.80	[0.50	1.30]	0.66^*^	[0.42	1.05]
Central	1.01	[0.64	1.60]	0.73	[0.46	1.14]	0.71	[0.45	1.11]
West	0.86	[0.47	1.59]	0.58^*^	[0.35	0.96]	0.57^*^	[0.35	0.92]
South	0.52^**^	[0.34	0.80]	0.39^***^	[0.26	0.57]	0.38^***^	[0.25	0.59]

Source: Authors’ estimates from NSSO survey, 2014

^a^Based on 3910 cases, ^*^significant at 10% level, ^**^significant at 5% level, ^***^significant at 1% level

## Discussion

Accidental injury is one of the important causes of deaths in India. There has been a sharp increase in cases of accidental injury due to heavy industrialization and a disproportionate rise in the number of vehicles [30]. As per our information, this study is a pioneering attempt to explore various socio- economic and demographic covariates of catastrophic expenditure on accidental injury in India. This study indicates the catastrophic economic impact on households due to accidental injury. Expenditure under all heads i.e., both direct and indirect costs, has provided a wide-ranging perspective on the cost associated with accidental injury. We have used data on household expenditure rather than annual income, to determine the nature of catastrophic expenditure by using 5%, 10% and 15% threshold levels out of the total household expenditure. In literature, when total expenditure is used as a denominator, most widely used threshold is 10% [31–34]. It shows an approximate threshold at which the household is forced to sacrifice their other basic needs and are forced to sell their productive assets, resort to borrowings and become impoverished [35]. We have used binary logistic regressions to examine the intensity of catastrophic payment due to accidental injury.

In the present study, nearly half of the sampled population who suffered from accidental injuries was in the age group of 30–59 years (48%). Various studies from India and across the globe has also noted similar finding in their studies [11, 36–39]. Various reasons can be cited for the higher number of accidents among the above-mentioned groups of the population. This age group represents the most productive group, which contributes significantly to the income of the households or the family [34, 40]. Accidental injuries significantly affect more to the productive age group between 30 and 59 years [13, 14, 41, 42]. However, fragility contributed little to the excess accidental injury risk under the age of 30–59 years population [43].

Similarly, males were more prone to accidental injury as compared to females. Males are more vulnerable to accidental injuries, and are more often the victims of accidental injury in comparison to females [44, 45]. Females were involved in fewer road accident deaths as compared to their male counterparts [46, 47]. Also, as men are often the sole income earners in many families, they have to travel often. Women may typically not be in jobs that have a higher risk of accidental injury. The other reasons could be that males are more aggressive and careless than their female counterparts [11, 39]. Generally females are considered to be risk averters and are more cautious. Males are also more mobile due to their job profiles and sometimes are the sole breadwinners in the family, resulting into higher number of road fatalities, and in turn higher catastrophic spending at all the threshold levels i.e., 5%, 10% and 15%. Women seem to be less associated with accidental injury, as women mostly walk, do not hold a valid driving license and have lower participation in the workforce [48, 49].

It was also observed that percentage share of accidental injuries in the rural areas was comparatively higher than urban areas. Rural areas had more road fatalities (56%) than urban areas (44%). They lack in terms of basic infrastructural facilities such as, roads, traffic lights and traffic operators. The accidents are more severe, resulting into higher casualties in rural locations [11, 23, 50]. Rural areas also lack in terms of basic facilities to treat emergency and serious cases of accidental injuries. In case of severe accidental cases or emergencies, referral is provided to the patients to the nearest city hospitals after providing basic first aid treatment [47, 51–53]. Inadequate or non-availability of the health infrastructure causes higher catastrophic spending to the households at all threshold levels (5%, 10% and 15%).

Majority of the population sought care from private facilities, were not insured, and even if insured, did not receive reimbursement from the insurance providers [40, 54]. More than half of the population sought care from private providers. Reasons can be cited in terms of availability of better infrastructure and proper care in the private facilities as compared to the government units. Studies also indicate that casualties are more in public hospitals in comparison to private facilities [55, 56]. This may be due to the infrastructural lags which are either not properly functional or absent in public facilities. Accountability can be another cited factor for the larger number of deaths in public facilities. The problem arises when these super specialty public hospitals, which are the last hope for the poor and helpless rural families, are totally mismanaged. Regarding the coverage of accidental insurance, in developing countries like India, more than 96% of victims of road fatalities have no definable source of health insurance at the time of accident [57]. Limited number of studies on community-based health insurance in LMICs also shows that lack of insurance can lead to higher level of OOPE and result into higher CHE [15, 27, 58, 59].

The study shows a direct and proportional relationship between spending on accidental injury and age. The socio-economic differentials in the level of OOPE indicates that spending on accidental injury was higher among the elderly population i.e., in the age group of 60+ population, and least among the less than 15 years of age group. CHE was higher for the elderly population, perhaps due to various reasons due to health conditions deteriorating in the older age and recovery from the accidental injury being a lengthy process. Elder population is also more prone to the accident related deaths and in many instances also suffers from post-accidental disabilities as compared to others [60, 61]. As per a study, drivers aged above 60, showed serious accidental injury rates twice as high as those of the 30–59-year-old group [43]. Various probable reasons has been cited such as age related physical mobility, ability to manage complex situations such as, at the intersections, turning out of the parking spaces, vision related difficulty especially in nights [62–64].

Gender was an important determinant of spending on accidental injury as spending among males was seen to be higher than females [65]. Literature also indicates that accidents involving males are more serious and harmful. Often, males are the sole earners in many families, so injury related disabilities may hamper the overall economic situation of the family [41, 66]. Similarly, those who were either formally educated or having education up to graduation or above, were spending more on accidental injury than others. It may be due to better awareness of road traffic laws and regulations, knowledge of traffic signs, or related fines [47, 67, 68]. However, our experience in the Indian context seems to show that impact of education on accidental injury is still a debatable issue. Some studies also indicate that road traffic-related knowledge was not correlated with formal education [69, 70]. So, a general conclusion cannot be drawn from this study and more studies are required to establish this fact.

The spending was more on accidental injury in the urban area. Literature also highlights that the density and severity of crash accidents was higher in the urban areas than the rural, which may result into higher spending [50, 71]. Other factors may also aggravate the spending on accidental injuries such as, better facilities and higher cost of treatment. Other studies also support our finding that the spending on accidental injury in the urban areas was comparatively higher than the rural areas [52, 72]. However, level of CHE was higher in the rural areas as compared to urban. The gap between rich and poorer section of the households was significantly higher, as the richer section of the population was spending a considerably higher amount on accidental injury than their counterparts from other categories [22, 73]. Poorer people are unable to afford costly treatment procedures and they avail care from public facilities; else, they have to endure distress financing [74]. A few studies also indicate some interesting findings such as, people from the higher income group may assign a higher priority to the perceived value of time and comparatively lower weight to the real cost or fines. Richer or richest individuals may, therefore, drive faster, which would increase their chances of being involved in an accident [73].

Accidental injury care from private facilities is quite expensive and unaffordable. Sometimes private centers lengthen the duration of stay in the hospital, resulting in higher level of OOPE for people. Another notable finding of the study was, as the length of hospital stay increases, the expenses incurred for the treatment also rises. Other studies have also shown similar results, which may be due to the difference in the severity of accidents or type of hospital [75]. There is a clear cut private and public divide in terms of OOPE [52]. Present study also supports the general notion that generally treatment for the accidental injuries in private hospitals involves high household OOPE and CHE as compared with public hospitals. Our study indicates that the cost of treatment for accidental injury in private hospitals was four times higher than public providers. The level of CHE was significantly higher in the private facilities. There was huge iceberg in terms of cost and in the treatment procedures of the public and private hospitals. In spite of these cost differentials there is a general opinion that RTA cases are better managed in private health care facilities. In case of lack of ability to pay, poorer section of the households admits the patients in the government hospital. However, they still need to pay a lump sum amount from their income, which may affects their daily way of living in many ways [75–77]. Deaths due to road casualties were very less and maximum people survived from these injuries. Those who survived accidental injury incurred a lower level of OOPE than the deceased. Literature also shows that [78, 79] the cost of inpatient care for the decedents was higher in comparison to that of survivors. Insured population incurred a higher level of OOPE than the uninsured population on the treatment of the accidental injury; so, merely having access to insurance was not helpful in reducing OOPE. No doubt, those who received reimbursement from the insurance were in a better position, as level of OOPE was comparatively lower for them. However, in the Indian context, only a handful of the population has access to any sort of insurance coverage. A large segment of the uninsured population is unable to manage the expenditure and, is forced to take discharge from the care units. In both ways they have to bear double burden either in form of wage loss if they choose rest/care and cost to avail the healthcare facility or else they have to compromise with the wages they could have earned in perfect functional/physical condition. All these factors further increase the economic burden on accidental injury cases and on their families [80].

Major source of financing for accidental injury was from the income or savings of the households. The levels of OOPE and CHE significantly increase in the case of road fatalities. Those who have resorted to borrowings were incurring higher OOPE, resulting into catastrophic spending and impoverishment. Because of this added expenditure, households are forced to opt for borrowings or taking loans to manage the shortfall. Spatial distribution of accidental injury shows a higher concentration of the cases in the southern states, followed by central and east. However, the levels of OOPE and CHE were higher in the western, central and southern regions in comparison to the other parts of the country. Indian Roads are getting more vulnerable beyond any surprise due to increase in motorization, accompanied by urbanization, and higher population density [59, 67, 81]. Various reasons can be cited such as tremendous growth in the level of income, GDP, industrialization and economic development and population density in these states [82, 83]. Increased urbanization and population growth causes an increase in pedestrian activity often accompanied by higher pedestrian fatalities [84, 85]. This observation is consistent with our findings.

Binary logistic regression analysis also shows similar trends in OOPE and CHE. Level of CHE was lower for females, illiterate households, Muslims, poorest section of the population, in public facilities, for insured population, shorter stay duration (1–2 days), using savings/income or other sources for financing accidental injury, and for the southern regions. The CHE increases for the elderly population aged 60+, males, uninsured population and for those who seek private care and also resort to borrowings as a source of financing.

## Conclusion

Indian healthcare system is the most privatized system where people still prefer big and private hospitals for trauma and accidental injury care. Due to the existence of unofficial dual healthcare delivery system in India, supply of the services depends upon the economic status of the population. Infrastructural issues such as negligence, understaffing, availability of equipment’s and manpower are the major reason behind increased casualties, and poor delivery of healthcare facilities in case of accidental injury. There is an immediate need to strengthen the public healthcare system and proper regulation is required for bringing the private healthcare sector into a well-regulated national health-care system. Special trauma care units can be set in the rural areas and can be managed appropriately to reduce the deaths and injuries from RTA. Proper awareness should be provided for avoiding accidental injuries. This can be done through mass campaigning and media promotion programs. Special target should be laid on the group of males in the age group of 30–59 years who are more prone to accidents. Safety measures should be adopted while using any type of vehicle on the roads. There is also immediate need to relook at the existing legal penalties for those who drink and drive or use mobile phones while driving. Factors responsible for regional variations in the accidental injury can be further examined in the Indian context. Majority of the people avoid renewal of third party insurance of the vehicles which later on proves to be detrimental for both health and financial situations. There should be some type of reinforcement measures for rechecking of third party insurance for vehicles.

The resultant financial burden caused due to RTA or accidental injury on the households is so huge that it cannot be ignored and little has been said and done to anodyne this pain. Hence, there is an urgent need for affordable health insurance policies to be made available by the government for the economically weaker sections, and also increased awareness among people regarding the availability of the insurance facility. This study provides further evidence for the need to strengthen the public health system in India in order to reduce the burden of OOPE on households caused due to accidental injury. Disabilities caused due to accidental injuries may affect adversely the individuals, households and overall economy. To confirm some of the findings (role of education), more evidences are required at micro level to find out how education influences the risk attitude, exposure and knowledge.

## Appendix 1

**Table 5:** Average medical expenditure (INR) per hospitalization case for each broad ailment category in different types of hospitals

Broad ailment category	Average medical expenditure (INR) per hospitalization case		
	Public	Private	All
Cancer	24,526	78,050	56,712
Cardio-vascular	11,549	43,262	31,647
Genitourinary	9295	29,608	24,525
Psychiatric and neurological	7482	34,561	23,984
Injuries	6729	36,255	23,491
Musculoskeletal	8165	28,396	21,862
Gastro-intestinal	5281	23,933	17,687
Ear	6626	19,158	15,285
Endocrine, metabolic and nutrition	4625	19,206	14,117
Blood diseases(including anaemia)	4752	17,607	13,313

Source: NSS KI (71/25.0): Key Indicators of Social Consumption in India: Health, 2014–15

## Appendix 2

**Table 6:** Prevalence of accidental injury by selected socio-economic and demographic profile, India, 2014

Co-variates	N (Persons with Accidental Injury)	N (Overall member profile)	% (Accidental Injury)
Age			
Less than 15	482	98,858	0.49
15–29	985	94,021	1.05
30–59	1934	114,223	1.69
60+	654	28,397	2.30
Sex			
Male	2973	170,195	1.75
Female	1082	165,304	0.65
Education level			
Illiterate	941	102,994	0.91
Up to primary	1011	93,847	1.08
Up to secondary	1509	106,857	1.41
Graduation and above	449	29,399	1.53
Residence			
Rural	2264	190,904	1.19
Urban	1791	144,595	1.24
Caste			
SC/ST	1111	99,306	1.12
Other Backward Classes (OBC)	1595	134,504	1.19
Others	1349	101,689	1.33
Religion			
Hindu	3227	253,816	1.27
Muslim	493	50,525	0.98
Others	335	31,158	1.08
Household size			
1–3 member	853	38,094	2.24
4–6 members	2335	180,736	1.29
7 + members	867	116,669	0.74
Wealth quintile			
Poorest	608	83,223	0.73
Poorer	845	51,073	1.65
Middle	788	71,355	1.10
Richer	935	63,951	1.46
Richest	879	65,861	1.33
Insurance coverage			
Not covered	3098	282,868	1.09
Covered	812	50,234	1.61
Survival status			
Alive	3910	333,104	1.17
Dead	145	2395	6.05
Region			
North	628	53,115	1.18
Northeast	312	41,174	0.75
East	697	60,753	1.14
Central	909	72,793	1.24
West	531	44,725	1.19
South	978	62,939	1.55
Total	4055	335,499	1.21

## Abbreviations

CHE: Catastrophic health expenditure
HICs: High-Income Countries
LMICs: Low and Middle-Income Countries
NSSO: National Sample Survey Organisation
OBC: Other Backward Caste
OOPE: Out of pocket expenditure
RTA: Road Traffic Accidents
SC: Scheduled Caste
SES: Socio-economic Survey
ST: Scheduled Tribe
WHO: World Health Organization
YLL: Years of life lost

## Footnotes

1 “Injury” refers to damage to the body produced by energy exchanges that have relatively sudden discernible effects [3]

2 A road traffic injury is a fatal or non-fatal injury incurred as a result of a collision on a public road involving at least one moving vehicle [2].

3 Similar as accidental injury

4 Household out-of-pocket expenditureon health comprise cost-sharing, self-medication and other expenditure paid directly by private households, irrespective of whether the contact with the health care system was established on referral or on the patient’s own initiative [20]

5 Health spending is taken to be catastrophic when a household must reduce its basic expenditure over a period of time to cope with health costs, but there is no consensus on the threshold proportion of household expenditure [74]

6 An accident, also known as an unintentional injury, is an undesirable, incidental, and unplanned event that could have been prevented had circumstances leading up to the accident been recognized, and acted upon, prior to its occurrence [3].

7 It includes accidental injury, road traffic accidents (RTA) and falls.
